# Population Modeling Approach to Optimize Crop Harvest Strategy. The Case of Field Tomato

**DOI:** 10.3389/fpls.2017.00608

**Published:** 2017-04-20

**Authors:** Dinh T. Tran, Maarten L. A. T. M. Hertog, Thi L. H. Tran, Nguyen T. Quyen, Bram Van de Poel, Clara I. Mata, Bart M. Nicolaï

**Affiliations:** ^1^Faculty of Food Science and Technology, Vietnam National University of AgricultureHanoi, Vietnam; ^2^KU Leuven, BIOSYST-MeBioSLeuven, Belgium; ^3^KU Leuven, BIOSYST, Crop BiotechnicsLeuven, Belgium; ^4^Flanders Center of Postharvest TechnologyLeuven, Belgium

**Keywords:** tomato, biological age, fruit development, ripening, optimal harvest strategy, modeling

## Abstract

In this study, the aim is to develop a population model based approach to optimize fruit harvesting strategies with regard to fruit quality and its derived economic value. This approach was applied to the case of tomato fruit harvesting under Vietnamese conditions. Fruit growth and development of tomato (cv. “Savior”) was monitored in terms of fruit size and color during both the Vietnamese winter and summer growing seasons. A kinetic tomato fruit growth model was applied to quantify biological fruit-to-fruit variation in terms of their physiological maturation. This model was successfully calibrated. Finally, the model was extended to translate the fruit-to-fruit variation at harvest into the economic value of the harvested crop. It can be concluded that a model based approach to the optimization of harvest date and harvest frequency with regard to economic value of the crop as such is feasible. This approach allows growers to optimize their harvesting strategy by harvesting the crop at more uniform maturity stages meeting the stringent retail demands for homogeneous high quality product. The total farm profit would still depend on the impact a change in harvesting strategy might have on related expenditures. This model based harvest optimisation approach can be easily transferred to other fruit and vegetable crops improving homogeneity of the postharvest product streams.

## Introduction

Tomato, *Solanum lycopersicum* Mill, is a worldwide economic valuable and healthy crop with good nutritional properties (Kimura and Sinha, 2008). It continues to increase in importance for consumption as a fresh crop. During the past decades, the tomato production area in Vietnam has been increasingly expanding as tomato has become an important export crop. As a result, the farmer's income from tomato cultivation is four-fold higher than that from rice cultivation (Ta Thu Cuc, 2003).

Currently, there are two main types of tomato cultivars being cultivated in Vietnam: traditional heat sensitive cultivars and new heat tolerant cultivars (Ha, 2015). The latter are widely grown in the North of Vietnam where the farmer can grow them during both the winter and summer season. Among the heat tolerant cultivars, “Savior,” a plum tomato, is favored for its high yield, good appearance and popularity among consumers.

Postharvest losses of tomato are still huge in Vietnam (Genova et al., 2006) as farmers are unable to define the optimal picking time that ensures a good postharvest life of fruit (Moneruzzaman et al., 2009). In contrast to European greenhouse production systems, where ripe fruit are harvested selectively, in the open field production systems as applied in Vietnam the crop is typically harvested at once. There, are several anecdotal reasons for this practice. Some farmers might lack knowledge about the best harvest practice and the consequences it will have on the marketing potential of their product. In some cases the labor cost can be higher than the profits growers can make due to market saturation. Also when the weather goes bad farmers decide to harvest the whole crop at once.

Growers mostly decide on picking date based on fruit color and the time after anthesis. However, the actual time required from anthesis to reach full maturity can vary due to genetic and environmental differences (Klee and Giovannoni, 2011). Moreover, the currently used color based classification of tomato is discrete and subjective and does not take into account the biological variation within a batch of fruit. By harvesting the whole crop at once, some fruit are harvested too early failing to properly ripen while others are harvested too late becoming susceptible to handling damage in the supply chain. Furthermore, no facilities are available to grade the fruit after harvest allowing the heterogeneous batches to reach the market. Hence, there is an urgent need to optimize the harvesting strategy for tomato fruit without going immediately into technological solutions.

The variation in postharvest storage behavior can often be interpreted as the expression of the same generic product behavior, only the choice of time zero from which moment the individual fruit are being observed is randomly determined by the moment of harvest (Hertog et al., 2007a). The biological (or physiological) age of the individual fruit is defined as the age of the product relative to an arbitrary reference point. The biological variation at harvest can thus be interpreted in terms of variation in the biological age of the harvested fruit.

**Figure 1:**
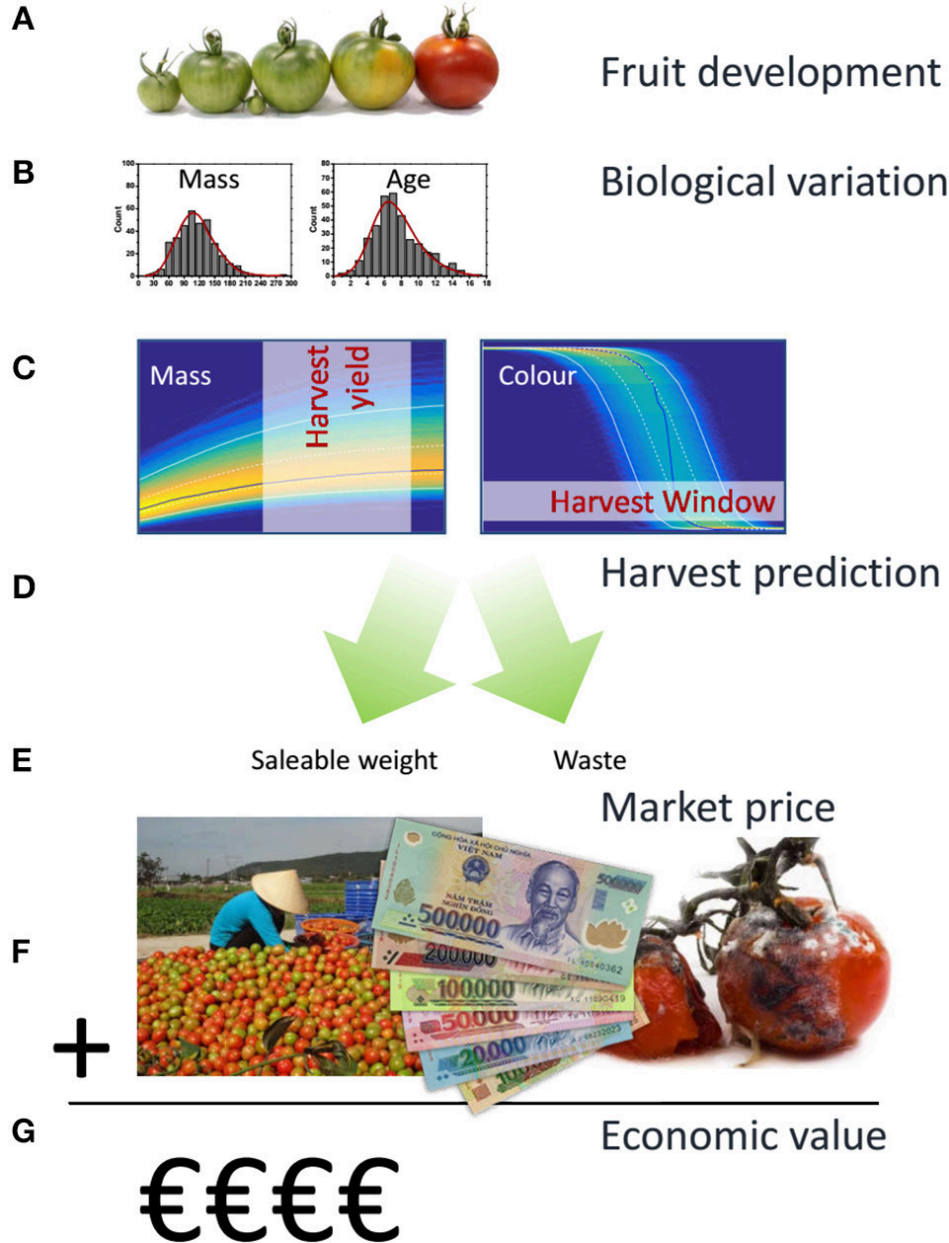
**Workflow applied in the current population model based approach**. Tomato fruit development can, from an economic perspective, be characterized by fruit growth and fruit color as they largely define yield and quality. In a first step **(A)** existing kinetic models were used to describe both aspects for individual tomatoes. Biological variation between individual fruit was subsequently characterized in terms of variation in final fruit mass and biological age **(B)**. Using Monte Carlo simulations the population dynamics for a large batch of fruit was calculated **(C)** generating the expected density distributions for fruit mass and color over time. Applying various harvesting strategies **(D)** total yield in the various color classes, including overripe waste, was determined from the Monte Carlo results **(E)**. Using an independent surface response model describing wholesalers price as a function of the color composition of a batch of fruit **(F)**, the economic value of the harvested fruit was calculated **(G)**.

So far, there have been few research groups using the biological age concept to classify the maturity of different fruit organs such as tomato (Hertog et al., 2004; Van de Poel et al., 2012, 2014), nectarines (Tijskens et al., 2007; Rizzolo et al., 2009), cucumber (Schouten et al., 2004), kiwifruit (Jordan and Loeffen, 2013), and apple (Tijskens et al., 2008, 2009). In this study, the aim is to bridge the gap between pre- and postharvest by using the biological age concept to optimize harvesting strategies that are at the root of postharvest biological variation. This approach is applied to the case of tomato (cv. “Savior”) grown in Vietnam during both the winter and summer season to quantify the potential economic benefits of more dedicated harvesting strategies to the Vietnamese growers. To compare fruit quality of the harvested crop, market acceptance is used to translate fruit quality of the harvested crop into an equivalent economic value taking into account fruit-to-fruit variation. The economic value of the crop is thus defined as the maximum amount of money a specific actor, in this case a wholesaler, is willing to pay for the harvested crop. By focussing on saleable weight discarding overripe fruit, optimisation of growers' revenues will go hand in hand with reducing postharvest waste which is a major worldwide concern (Gustavsson et al., 2011). The workflow applied in this study is outlined in Figure 1. The innovation of this approach lies in the integration of existing concepts bridging the gap from pre-harvest horticultural production via postharvest quality back to the economic impact for the growers taking into account biological variation.

## Materials and methods

### Plant material

Tomato seedlings (cv. “Savior”) were transplanted during the winter season 2014 and the summer season 2015 at the Fruit and Vegetables Research Institute, Hanoi, Vietnam (21°00′38.9”N 105°55'39.2”E). Plants were grown under cover with protection against birds, wind, rainfall, and excessive sunlight. Shortly after anthesis flowers were labeled checking for new flowers three times at 5 d intervals. In total, 700 tomato flowers from 300 randomly chosen plants were labeled covering a wide range of fruit variation. From these labeled flowers 342 fruit grown in winter and 370 fruit grown in summer were successfully monitored for color and size on the plant. During fruit development measurements were taken at 3 d intervals while during fruit ripening measurements were taken at 2 d intervals.

### Experimental measurements

#### Fruit mass

Fruit diameter was monitored on-plant using a caliper (Mitutoyo, Japan). Fruit mass was calculated from fruit diameter assuming a spherical fruit and an average fruit density of 873 kg.m^−3^ according to:

$$\begin{array}{l} m=\frac{4}{3}\pi {\left(\frac{D}{2}\right)}^{3}d \end{array}$$

where *m*: fruit mass (kg); *D*: fruit diameter (m); *d*: fruit density (kg.m^−3^)

The constant value for fruit density was based on a preliminary experiment were both diameter and mass were measured on a range of harvested tomato fruit showing that the ratio between measured mass and volume calculated from the measured diameter could be considered constant over the whole fruit mass range (Figure S1)

#### Fruit color

Fruit color was always measured on the same spot at the equator using a Minolta CM-2500d colorimeter (Minolta Camera Co., Ltd, Osaka, Japan) and expressed in the CIELAB color space L^*^, a^*^, and b^*^. The fruit color was expressed as hue angle (degree).

$$\begin{array}{l} H=\text{arctan}\left(\frac{b^{*}}{a^{*}}\right) \end{array}$$

### Model development

#### Fruit model

During fruit development, fruit mass of the green fruit is gradually increasing until some maximum fruit size is reached. Subsequently the fruit will start to ripen as mirrored by its color change. To describe these changes the modeling approach developed earlier in our group was adopted (Van de Poel et al., 2012) which is summarized below.

The change of fruit mass (*M* (kg)) in time was modeled using the following differential equation describing the Gompertz growth model (Winsor, 1932):

(1) $$\{\begin{array}{l} \frac{d}{dt}M(t)=k_{m}M\ln\;\left(\frac{M_{\max}}{M}\right)\\ M(0)=M_{\max}\exp\left(-C\right) \end{array}$$

with *k*_*m*_ (d^−1^): the growth rate; *M*_max_ (kg): the maximum fruit mass; *C*: a dimensionless displacement factor from the Gompertz function. The parameters *k*_*m*_ and *C* are assumed to be constant for a specific cultivar, while *M*_max_ was assumed to be different for single every fruit.

Color change (measured as *H* in degree) was modeled using an exponential decay model implemented in its differential form.

(2) $$\{\begin{array}{l} \frac{d}{dt}H(t)=-\left(H-H_{\min}\right)k_{h}\\ H(0)=H_{0} \end{array}$$

with *k*_*h*_ (d^−1^): the rate of color change; *H*_min_ (degree): the minimum hue value; *H*_0_ (degree): the initial hue value. The parameters *k*_*h*_, *H*_min_, and *H*_0_ were assumed to be constant for a specific cultivar.

Color change was modeled as being triggered once the fruit approaches its maximum mass by incorporating a biological switch for the rate constant *k*_*h*_ following:

(3) $$\begin{array}{r} k_{h}=\frac{k_{h}^{\max}}{{\left(1+\left(\left(M_{\max}-M\right)/M_{\max}\right)\right)}^{s}} \end{array}$$

with *k*_*h*_^max^ (d^−1^): the maximum rate of color change once fully triggered; *s*: (dimensionless) defining the steepness of the switch.

#### Biological age

While the experimental time is counted relative to the first observed moment of anthesis, the individual fruits will all have a slightly shifted starting point as defined by their own biological age. This biological age of an individual fruit (*t*_*age*_ in d) can be calculated from the experimental time values (*t*_*exp*_ in d, relative to the day of harvest) by adding a fruit specific biological shift factor (Δ*t* in d) following Equation 4.

(4) $$\begin{array}{r} t_{\text{age}}=t_{\exp}+\Delta t \end{array}$$

### Model calibration using time series based data

The ODE based model was implemented and model parameters were estimated using OptiPa (Hertog et al., 2007b; www.optipa.be), a dedicated simulation and optimisation tool for ODE based models which was developed using Matlab (The MathWorks, Inc., Natick, MA, USA). The integrated model (Equations 1–4) was calibrated using the two dataset on fruit color and mass collected during the winter and summer season. Based on the data, common values for *k*_*m*_, *C*k_*m*_, *k*_*h*_^max^, *H*_min_, and *H*_0_ and fruit specific values for *M*_max_ M_max_ M_max_and Δ*t* were estimated. During the least square non-linear regression, the residual sum of squares was calculated by comparing the simulated values resulting from Equations 1–3 to the time corrected experimental values applying Equation 4. The dependent variables mass and color were both normalized (by subtracting their mean and dividing by their standard deviation) while calculating the combined residual sum of squares to give them equal weight during the model fitting. The ODE45 solver was selected for the numerical integration of the ODE based model.

### Price model

In order to judge market acceptance of batches of fruit of different homogeneity, fruit mixtures were presented to a panel of 30 wholesalers. To quantify acceptance, wholesalers were asked to judge the quality in terms of a price per kg for each mixture. The wholesalers were presented either homogenous batches of a single ripening stage (Figure S2), or heterogeneous batches following a mixture design as indicated in Table 1. The mixtures were created to mimic normal harvested crop. This evaluation was performed for both winter and summer tomatoes in one session. The prices were normalized between 0 and 1 per wholesaler and per season. (Table 1), making it possible to compare the relative prices of the various tomato mixtures between wholesalers and seasons.

**Table 1:** **Mixture design for different ripening stages (RS) of tomato cv. “Savior” grown in winter and summer**.

**Mixture^1^**	**Mixing ratio**						**Normalized price/ kg^2^**	
	**RS6**	**RS5**	**RS4**	**RS3**	**RS2**	**RS1**	**Winter**	**Summer**
1	1	0	0	0	0	0	1.00 ± 0.02^a^	1.00 ± 0.02^a^
2	0	1	0	0	0	0	0.98 ± 0.05^a^	0.97 ± 0.07^a^
3	0	0	1	0	0	0	0.93 ± 0.08^a^	0.73 ± 0.20^b^
4	0	0	0	1	0	0	0.51 ± 0.26^c, d^	0.61 ± 0.19^b, c, d^
5	0	0	0	0	1	0	0.36 ± 0.21^e^	0.30 ± 0.18^e^
6	0	0	0	0	0	1	0^f^	0^f^
7	0.25	0.25	0.25	0.25	0	0	0.63 ± 0.19^b, c^	0.62 ± 0.16^b, c^
8	0.2	0.2	0.2	0.2	0.2	0	0.54 ± 0.20^b, c, d^	0.52 ± 0.19^c, d^
9	0	0.2	0.2	0.2	0.2	0.2	0.49 ±0.21^d, e^	0.47 ± 0.20^d^
10	0.1	0.3	0.3	0.15	0.1	0.05	0.54 ± 0.18^b, c, d^	0.50 ± 0.18^c, d^
11	0.1	0.3	0.2	0.1	0.1	0.2	0.50 ± 0.16^c, d, e^	0.50 ±0.21^c, d^
12	0.1	0.5	0.4	0	0	0	0.66 ± 0.14^b^	0.62 ± 0.17^b, c^
13	0.1	0.2	0.2	0.2	0.2	0.1	0.47 ± 0.16^d, e^	0.48 ± 0.19^c, d^

*The mixtures were created to mimic normal harvested crop*.

1 *Tomatoes at different ripening stages were mixed at various ratios ranging from 0–1. RS1, mature green fruit; RS2, breaker fruit; RS3, light orange fruit; RS4, orange fruit; RS5, red fruit; RS6, red ripe fruit*.

2 *Average normalized price values accompanied by standard deviation were assessed by 30 wholesalers for winter and summer tomato*.

*Within a column, results with the same letter were not significantly different in a one way Tukey multiple comparison test on a 95% confidence level*.

The dependency of the price on composition of the batch in term of different ripening stages was modeled by applying a mixture design. In a mixture experiment, the independent factors (the ripening stages) are proportions of different components of a blend together summing up to 100%. Under the assumption that the presence of extremely different maturity classes within a batch could potentially interact with each other in negatively affecting the overall price, only interaction terms were included for the most different maturity classes. (Equation 5):

(5) $$\begin{array}{r} Y=\alpha_{1}RS_{1}+\alpha_{2}RS_{2}+\alpha_{3}RS_{3}+\alpha_{4}RS_{4}+\alpha_{5}RS_{5}+\alpha_{6}RS_{6}\\ +\alpha_{7}RS_{1}RS_{6}+\alpha_{8}RS_{2}RS_{6}+\alpha_{9}RS_{1}RS_{5} \end{array}$$

where *Y* is the response variable (normalized price/kg); α_1_−α_6_ are regression coefficients for the main linear effects, α_7_−α_9_ refer to the interaction effects. *RS*_1_–*RS*_6_ represent the independent variables being the percentage of fruit in the batch representing different ripening stages ranging from immature green (*RS*_1_) to ripe red (*RS*_6_). Note that the model, being a mixture design, does not include an intercept term due to the correlation between all the components (their sum equals 100%). The ripening classification based on Hue limits is given in Table S1. The coefficients were estimated by least square non-linear regression. The significance of the overall model and of each coefficient was evaluated by analysis of variance (ANOVA). The statistical analysis were done using JMP® Pro 12, SAS Institute Inc., Cary, NC, 1989–2015.

### Optimizing harvest strategy for tomato

**Figure 2:**
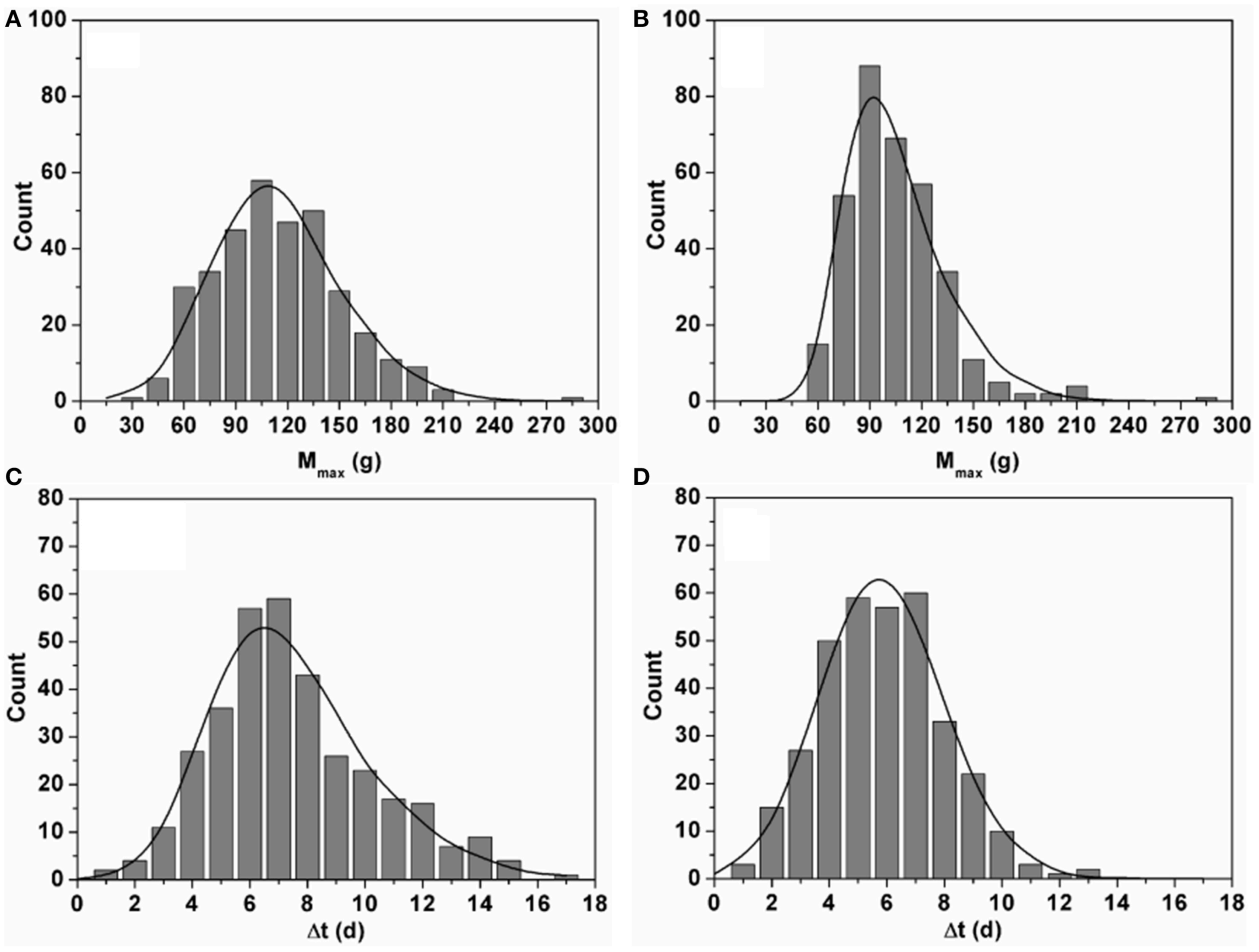
**Histograms showing the distributions of estimated M_max_ (g) (A,B)** and Δt (d) **(C,D)** for the 342 fruit grown in winter **(A,C)** and the 370 fruit grown in summer **(B,D)**. The curves represent the equivalent distributions based on 10,000 fruits generated during the Monte Carlo analysis. Comparing the two shows the agreement between the experimentally observed variation and the variation mimicked during the Monte Carlo simulations.

**Figure 3:**
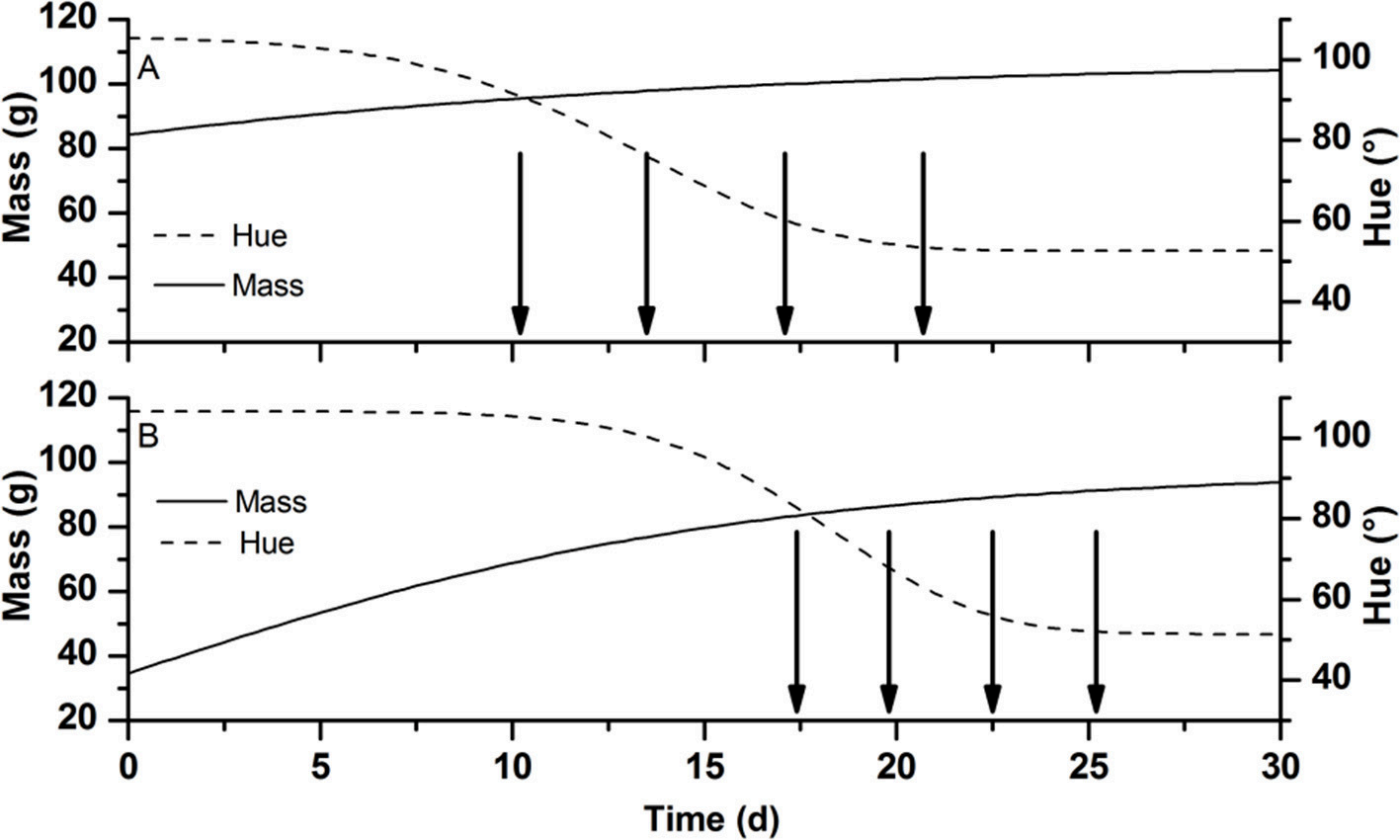
**Schematic representation showing dynamic harvest strategy for tomatoes at RS5 and RS6 grown (A)** in winter and **(B)** in summer. The lines represent the typical change in mass in g (full line) and hue color in degree (broken line) of developing tomato fruit during the winter and summer season. Arrows indicate the planned moments of harvest. Time 0 is taken at an arbitrarily early data well before the colouration of the fruit skipping most of the fruit growth part to focus on the period near harvest.

To find the optimal harvest strategy at which the maximum price was realized a Monte-Carlo analysis was performed using the OptiPa software. Starting from the fruit specific parameters *M*_max_ and Δ*t* as derived from the calibration data a new virtual parameter set was generated representing a population of 10,000 tomatoes with the same distribution characteristics (average, variation, shape, and correlation) as the original parameter set (Figure 2). In combination with the other cultivar specific model parameters, these were used to simulate the fruit growth model 10,000 times generating detailed time varying distributions for both fruit color and fruit mass. The 10,000 Monte Carlo simulation were analyzed using custom Matlab scripts basically counting the number of fruit falling in the various ripening stages at any point in time during the simulation. Using these scripts, fruit meeting the harvest criteria were virtually harvested while the remaining fruit was allowed to continue to develop. The color distribution of the harvested fruit was translated into its equivalent economic value using the price model taking into account production volume based on the simulated fruit weight. Overripe fruit (defined as having a hue color <54°) was considered waste and would not contribute to the overall production. Harvested volumes from the subsequent harvest dates were cumulated to obtain the total economic value generated. Different harvest strategies for tomato grown in both winter and summer were simulated to find the scenario with the best return and the lowest postharvest waste. These strategies representing either a single harvest (all tomatoes in the field were harvest at once) or focussed multiple harvests (only harvesting ripening stages RS4, RS5, and RS6 or only RS5 and RS6) combined with fixed harvest intervals (one, two, three or four day intervals) or flexible harvest intervals (dynamic harvest). The dynamic harvest regime consisted of multiple harvests of ripening stages RS5 and RS6 at varying time intervals as indicated in Figure 3.

## Results

### Modeling fruit development

**Figure 4:**
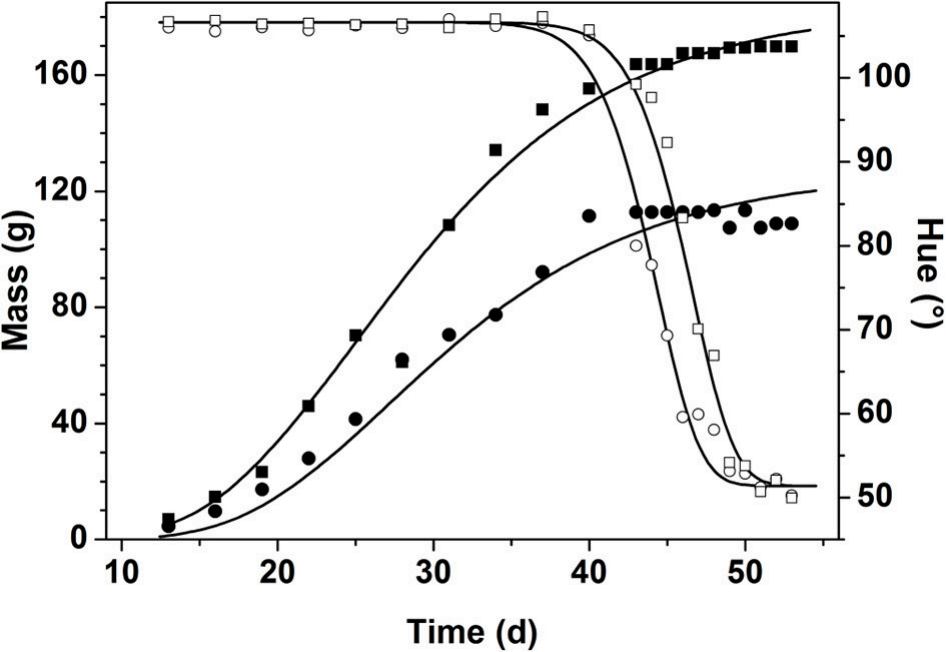
**Change of fruit mass in g (closed symbols) and hue color in degree (open symbols) during fruit development and ripening for two randomly chosen fruits from the first flowering period in winter**. The symbols represent the measured data while the lines represent the model fit for the selected fruit.

The typical change of mass and color during fruit development and ripening is illustrated by two exemplar fruits in Figure 4. All fruit followed an identical growth pattern. The development stage for “Savior” took about 52–55 d after anthesis. It was observed that different fruit reached a wide range of mass (from 50 to 110 g) despite their similar flowering time.

During the main part of fruit growth fruit color remained constant. Color change was only triggered once the fruit approached its final mass. While mass remained almost constant, color dropped from immature green (hue ranging from 104 to 106°) down to mature red (hue ranging from 50 to 55°). Moreover, the color data revealed a shift along the time axis between fruit, indicating the variation in biological age between the individual fruit.

Using both mass and color data from the time series obtained in winter and summer, the integrated model (Equation 1–4) was calibrated by estimating the various model parameters through non-linear regression analysis. The generic parameter estimates are given in Table 2. The variation in final fruit mass and time shift was captured by the fruit specific model parameters *M*_max_ and Δ*t* which were estimated for every single fruit. The distribution of the fruit specific parameters is shown in Figure 2. The maximum fruit mass *M*_max_ for winter ranged from 25.5 to 275.2 g with a mean of 107.1 ± 36.9 g and for summer ranged from 48.9 to 275.7 g with a mean of 99.03 ± 28.39 g, representing the broad range of fruit mass encountered. The mean values of Δ*t* for winter and summer were 7.01 ± 2.82 d and 5.39 ± 2.12 d, respectively.

**Table 2:** **Parameter estimates for the calibration of the integrated model (Equations 1–4 fitted to the dataset of mass and color of tomato grown in winter (*n* = 342) and summer (*n* = 370)**.

**Parameter**	**Winter (*R*^2^ = 98.9%)**	**Summer (*R*^2^ = 98.5%)**
***GROWTH MODEL PARAMETERS***		
*C*	4.87 ± 0.03	9.76 ± 7.26·10^−4^
*k_*m*_* (d^−1^)	0.0702 ± 0.0003	0.11 ± 4.57·10^−5^
***COLOR CHANGE MODEL PARAMETERS***		
$k_{h}^{max}$ (d^−1^)	26.02 ± 2.45	14.83 ± 0.55
*H_*o*_* (°)	105.74 ± 0.04	106.63 ± 0.05
*H_min_* (°)	52.55 ± 0.20	51.37 ± 0.07
***BIOLOGICAL SWITCH PARAMETER***		
*S*	54.30 ± 0.88	33.39 ± 0.30

*The parameter estimates are accompanied by their standard errors*.

*C: A dimensionless displacement factor from the Gompertz function; k_m_ (d^−1^): The growth rate; $k_{h}^{max}$ (d^−1^): The maximum rate of color change once fully triggered; H_min_ (°): The minimum hue value; H_0_ (°): The initial hue value; s: (dimensionless) defining the steepness of the switch*.

### Price model describing the market value of the harvested crop

To examine how the economic value changed as a function of the heterogeneity of the harvested crop representative fruit mixtures were judged on their economic value by 30 wholesalers. Pure homogeneous batches of either RS1 (green) or RS6 (fully ripe) where positioned at the two extremes of the normalized price spectrum ranging from 0/kg to 1/kg (Table 1).

For both seasons a batch of tomato containing only RS6 (mixture N°1) or RS5 (mixture N°2) had the highest prices (0.99/kg and 0.98/kg respectively). For winter tomatoes, there was no significant difference in price given for a batch of only RS3 (N°4) and mixtures of equal percentage of RS6 to RS2 (N°8) or of RS1to RS5 (N°9), and combination of all ripening stages (N°10, 11, 13). The price of a mixture containing 25% of each stage from RS6 to RS3 (N°7), was not statistically significant from that of a mixture containing 10% RS6, 50% RS5, and 40% RS4 (N°12).

**Figure 5:**
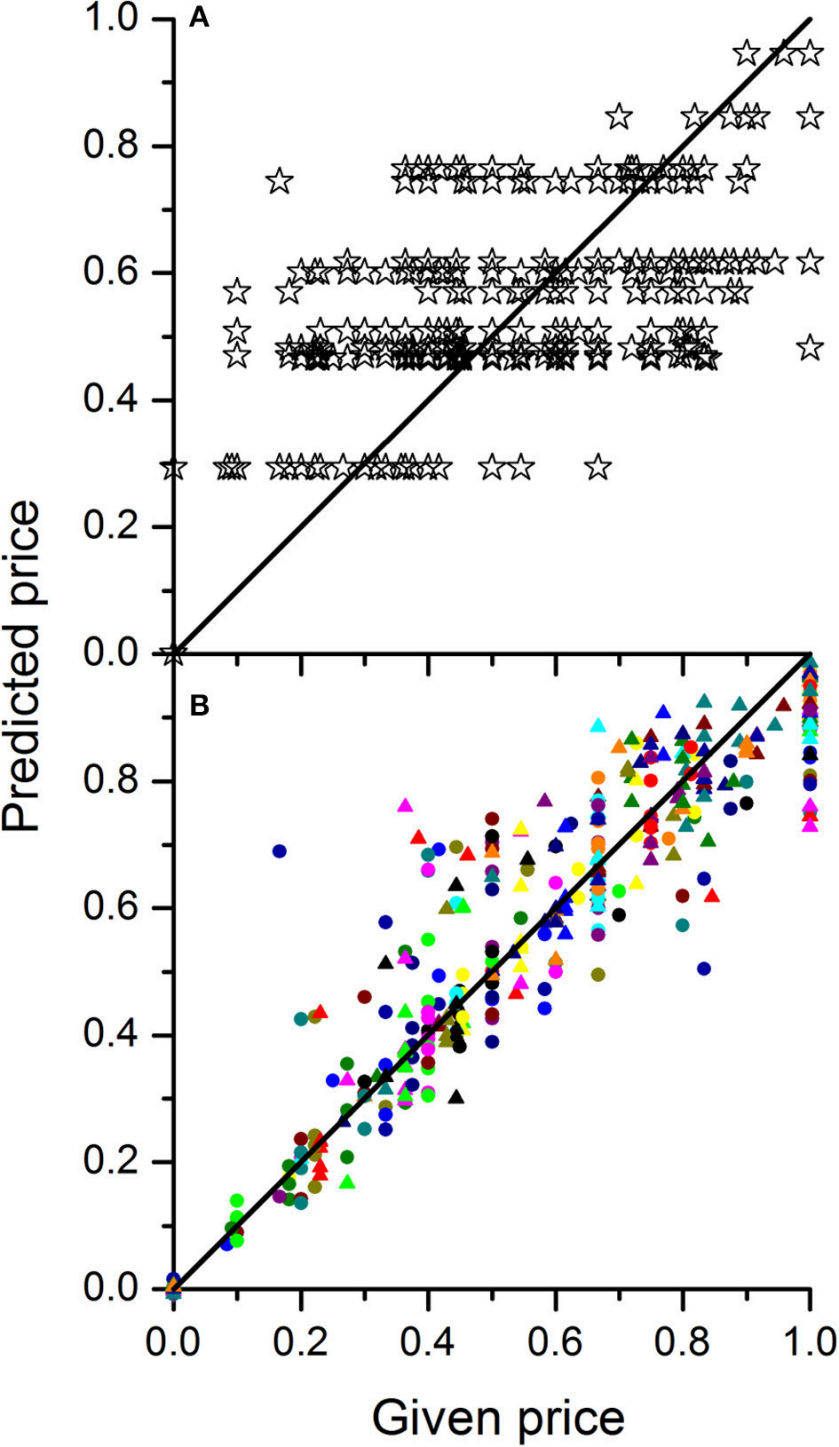
**Plots of predicted normalized price vs. the given normalized price for tomato grown in summer either based on the calibration of a single mixture model to the combined data of 30 wholesalers (A)** or by calibrating one mixture model per wholesaler **(B)**. The different colored symbols in B represent the 30 different wholesalers. In case of a perfect price model calibration, all points should sit on the diagonal line.

In order to investigate the dependence of price on the composition of the batch the linear regression model from Equation 5 was fitted to the data combined over all 30 wholesalers. The parameter estimates for winter and summer tomato are given in Table 3. The explained part was 0.64 and 0.63 for winter and summer tomatoes respectively (see Figure 5 for the summer fruit results). When the responses of the wholesalers were analyzed per wholesaler, explained parts for the individual wholesalers ranged from 0.51 to 0.97 with an average explained part of 0.88 and 0.87 for respectively summer and winter fruit (see Figure 5 for the summer fruit results).

**Table 3:** **Parameter estimates of the price mixture model fitted to the combined responses of 30 wholesalers**.

**Explanatory variables**	**Model parameters**	**Estimates**	***p*****-value**
**WINTER SEASON**	***R*****^2^ = 64 %**		
RS1	α_1_	0.00 ± 0.03	1.00
RS2	α_2_	0.35 ± 0.03	<0.0001
RS3	α_3_	0.47 ± 0.03	<0.0001
RS4	α_4_	0.81 ± 0.03	<0.0001
RS5	α_5_	0.84 ± 0.03	<0.0001
RS6	α_6_	0.94 ± 0.03	<0.0001
RS6 × RS1	α_7_	−3.21 ± 2.90	0.27
RS6 × RS2	α_8_	−4.11 ± 0.88	<0.0001
RS5 × RS1	α_9_	−0.21 ± 0.88	0.81
**SUMMER SEASON**	***R*****^2^ = 63 %**		
RS1	α_1_	0.00 ± 0.03	0.98
RS2	α_2_	0.29 ± 0.03	<0.0001
RS3	α_3_	0.57 ± 0.03	<0.0001
RS4	α_4_	0.62 ± 0.03	<0.0001
RS5	α_5_	0.85 ± 0.03	<0.0001
RS6	α_6_	0.95 ± 0.03	<0.0001
RS6 × RS1	α_7_	−2.33 ± 2.86	0.42
RS6 × RS2	α_8_	−3.66 ± 0.86	<0.0001
RS5 × RS1	α_9_	−0.10 ± 0.87	0.90

*The parameter estimates are accompanied by their standard error and p-values. p-values below 0.05 indicate estimates not significantly different from zero*.

### Monte carlo evaluation of harvesting strategies

**Figure 6:**
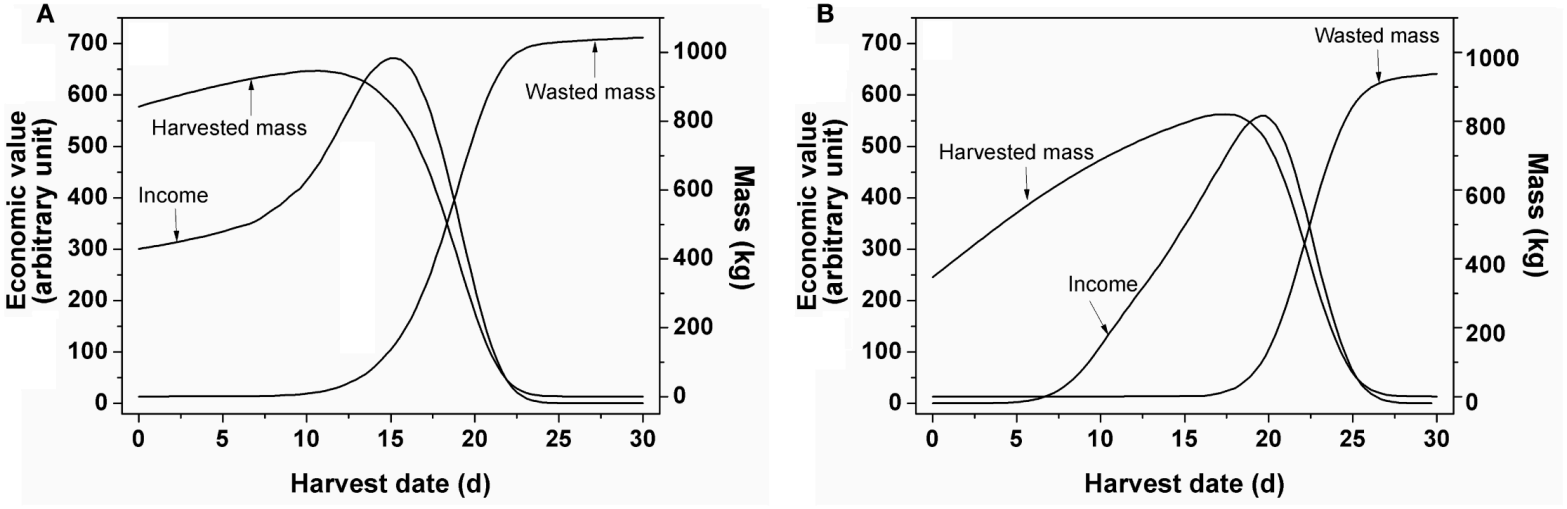
**Changes in economic value, harvested and wasted mass as function of time by single harvest for tomato grown (A)** in winter and **(B)** in summer as extracted from the Monte Carlo analysis. Based on 10,000 simulated fruits a 30 d harvest window was considered. For each day counts were generated for the number of fruit falling in the various ripening classes while keeping track of the total harvested fruit mass. Overripe fruit was assigned to waste. Based on the resulting mixture of ripening classes the economic value of the unsorted fruit was calculated.

In order to evaluate the various harvest strategies taking into account fruit-to-fruit variation, a Monte Carlo approach was applied. Figure 6 illustrates the situation in which a single harvest was applied. Considering a 30 d harvest window the highest economic value for winter tomatoes was observed for a harvest at 15 d while that for summer tomatoes was about 5 d later (19.8 d). The figure shows the realized economic value and the harvested and wasted biomass as function of time assuming all fruit was harvested during a single harvest. The normalized value increased with the increasing harvested mass. When the total harvested mass started to decrease, its economic value continued to increase for a little longer as the maturity of the diminishing amount of harvested fruit continued to increase. Only when the harvest was further delayed the economic value started to decrease as well. This coincided with an increasing amount of waste accumulating as fruit became overripe. Eventually all tomatoes would be harvested overripe, reducing the value to zero.

**Figure 7:**
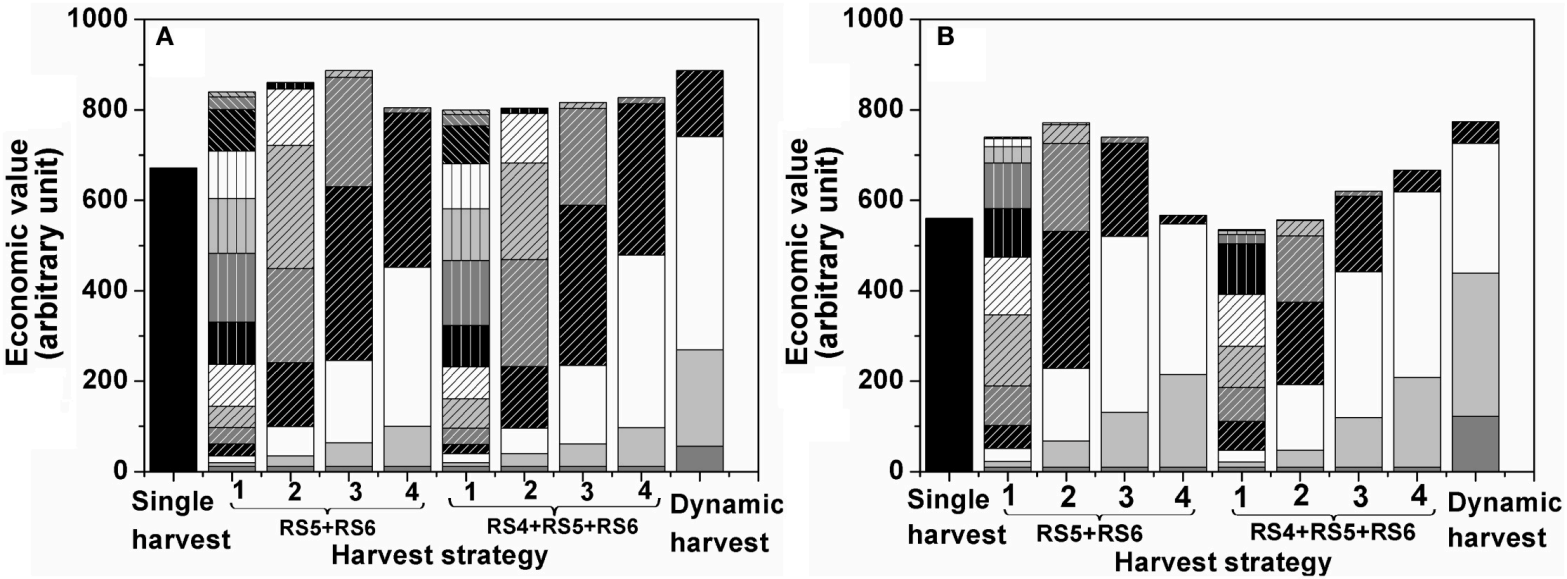
**Optimal harvest strategy by multiple harvests for tomato grown (A)** in winter, **(B)** in summer. Each bar represents a different harvest strategy. The leftmost bar refers to a single harvest were all fruit are harvested at once. The next 4 bars refer to harvests where only RS5 and RS6 were harvested. The next 4 bars refer to harvests where only RS4, RS5, and RS6 were harvested. The numbers 1–4 refer to the interval period between two harvests ranging from one to four day harvest intervals. The rightmost bar refers to the dynamic harvest: flexible harvest intervals as outlined in Figure 3 harvesting only RS5 and RS6. The different patterned parts of a bar represent the economic value generated per harvest day under the various strategies with the bottom part of each stack representing the first harvest day and subsequent layers referring to the subsequent harvest days.

To study the effect of harvest interval and the maturity classes targeted during harvest on economic return, multiple harvest strategies were simulated. In the Monte Carlo simulation tomatoes were harvested at either RS4, RS5, and RS6 or only at RS5 and RS6 applying harvest intervals ranging from1 d to 4 d (Figure 7). In addition, one dynamic harvesting scenario was simulated following the harvest schedule from Figure 3. For both winter (Figure 7A) and summer (Figure 7B) tomato the optimal single harvest from Figure 6 was taken as a reference. The additional harvest strategies all started earlier from the moment the first ripe fruit (RS6) would be on the vines (10.2 d for winter and 17.4 d for summer fruit). The different patterned parts of each bar represent the economic value generated per harvest day. The bottom part of each stack represents the first harvest day with subsequent layers referring to subsequent harvest days. The height of each bar represents the accumulated economic value over the whole harvest period for a given harvest strategy.

## Discussion

### Fruit-to-fruit variation was accurately captured by the fruit model while revealing seasonal effects

The observed growth curves (Figure 4) can be understood in terms of the known underlying fruit development processes (Gillaspy et al., 1993). The high variation in final fruit mass (Figure 2) can be explained by the fact that fruit are exposed to different microclimate conditions and sink/source relations within the plant (Van de Poel et al., 2012) which is not related to the biological age of the fruit as such.

The changes in fruit color can be understood in terms of the breakdown of chlorophyll and the production of carotenoids, two processes that occur in parallel (Fraser et al., 1994). While the model assumes a single constant final color value for all fruit, small fruit to fruit variation does exist. Although one might expect some correlation between this final color and final fruit mass this was not the case (Figure S3A). Data in Table 2 reveal that the generic parameters were estimated very accurately for both seasons as demonstrated by their small approximate standard deviations. When the generic parameters were compared between winter and summer tomatoes, some interesting trends were observed. While the growth parameters (*C* and *k*_*m*_) of winter tomato were almost half of those of summer tomato, the rate of color change $k_{h}^{max}$ of the former (26.02 d^−1^) was double the latter (14.83 d^−1^), indicating that the high summer temperature stimulates the growth rate of tomato but slows down the color change and with that fruit ripening. At the end of ripening, they both have a similar value for *H*_min_, of about 52°. For *s*, the higher value obtained for winter (54.30) than for summer (33.39), implies that the color change of winter tomato is triggered more toward the end of the growth cycle as compared to the summer tomato which started to color earlier. Clearly, the Vietnamese growing season varies largely affecting the supposedly generic model parameters thus contrasting the assumptions from Van de Poel et al. (2012). However, the Vietnamese growing practices and climate conditions are completely different from the Belgian situation where tomatoes are grown almost year round under well controlled conditions inside Venlo type glass greenhouses. In the end, by introducing season specific parameter values the model could be applied successfully. Further, research is needed to quantify the extent to which these parameters vary over the years and seasons.

Though the fruit model does not pretend to be a detailed description of the physiological reality it does contain elements inspired by the fruit's physiology. Especially the biological switch is an empirical approach to simplify the underlying climacteric regulation of fruit ripening. In real life this is about the plant hormone ethylene orchestrating a complex cascade of events turning on the various processes involved in fruit ripening (Lin et al., 2009). In spite of being descriptive in nature the model is fit to purpose and convenient to capture biological variation as observed through the fruit specific model parameters. The estimated values for Δ*t* and *M*_max_ showed no correlation, indicating two distinctly different sources of biological variation were involved (Figure S3B).

### The price model revealed inconsistent behavior between individual wholesalers

The common harvesting practice in some Vietnamese regions is that farmers harvest their whole crop at once resulting in a mix of various ripening stages. This indirectly causes economic losses as some fruit are harvested overripe while others are still too immature to gain full profits. At the same mixture composition, the averaged normalized prices of tomato grown in winter based on the evaluation by 30 wholesalers were either higher or at least equal to those of tomato grown in summer (Table 1). This indicates that within the fixed normalized range a shift has occurred toward higher prices. This can be explained by the fact that the winter tomatoes have better overall appearance and fruit weight therefore increasing the normalized price relative to its extremes. When the more green tomatoes were added to the mixtures, a lower wholesale price was obtained as Vietnamese consumers do not have the habit to buy green tomato. Similar trends were observed for summer tomato.

Concerning the dependence of price on the composition of the batch of fruit Table 3 revealed that the parameter estimates for both seasons had similar magnitudes. They showed that the main factors had positive effect on the normalized price with the size of the effect increasing with maturity stage from about 0 (for RS1) to about 1 (for RS6). This range is a direct consequence of the normalization of the price data where the prices given by each wholesaler was rescaled between 0 and 1. Given the parameter estimate for RS1 was not significant mirrors the fact that the wholesalers were unanimously about RS1 representing the lowest economic value. When green fruit were added to the mixture, lower prices were obtained for the batch. This was due to the inherent lower prices paid for the more immature fruit as mimicked by the negative values obtained for the interaction terms. However, it was expected that the presence of more immature fruit stages would suppress the prices disproportionally. Although the coefficients for the various interaction terms were all estimated to be negative only one of them (RS6 × RS2) was statistically significant (Table 3). From a logical point of view one would expect that if the negative interaction term RS6 × RS2 is significant the presence of even more immature fruit should definitely have a significant negative effect as well (thus resulting in a significant negative term for RS6 × RS1). However, this could not be confirmed through the current experimental data which might indicate a difference in opinion between the 30 wholesalers. Also the relative low explained part indicates inconsistencies in how the wholesalers judged the various mixtures. When the responses of the wholesalers were analyzed per wholesaler much better results were obtained indicating that the limited fit of the price model is not due to restrictions of the model structure applied, but merely due to a lack of agreement between individual wholesalers on the economic value of the fruit.

To predict the market price for a given mixture of fruit one could either use the overall mixture model calibrated on all wholesalers as one or make multiple predictions using the individually calibrated models averaging out the predicted prices afterwards. Both approaches would eventually lead to the same results although the latter approach would allow to provide insight in the market uncertainties depending on who one would sell to.

### Model based evaluation of various harvesting strategies enables to quantify the economic incentive for growers to move away from their current practice

The Monte Carlo analysis from Figure 6 combined the model on fruit growth with the price model. Based on the simulation of fruit development of 10,000 individual fruit (with regard to mass and color), using the parameter distributions from Figure 2 as an input, the evolution of fruit color distribution and fruit mass was collated over time. Overripe fruit was assigned to waste while for the remaining batch of fruit the economic value was calculated using the price model with the parameters from Table 3. By applying a single harvest, the economic value of the crop depended on the level of heterogeneity of the harvested crop in relation to the amount of overripe fruit present. In addition, the maximum normalized economic value for winter tomato was higher than that for summer tomato because the fruit weight and the normalized price for the former were higher than for the latter (Figure 6).

Theoretically, maximum profit is obtained when only harvesting the most mature heaviest fruit (RS6). Of course, to prevent waste, fruit has to be harvest as often as needed, based on the time needed for the RS5 fruit to develop into RS6 before turning into waste. Depending on the season this might require 1 d or 2 d harvest intervals. By harvesting less frequently, workload can be reduced but one should at the same time prevent waste to accumulate as this would imply economic losses. As a first alternative to a single harvest, fruit harvest was simulated for a narrow maturity range of RS5–RS6 varying the harvest interval from1 d to 4 d (Figure 7). For the slower growing winter fruit the economic value increased with an increasing harvest interval. The 1 d intervals resulted in many small harvests while, in between two harvests, it did not allow enough time for the remaining fruit to ever develop into full ripe fruit of RS6. By increasing the harvest interval to 3 d more time is available for the fruit to continue to develop in heavier ripe fruit without turning into waste increasing the obtained market price and minimizing waste at harvest. Going from 3 d to 4 d interval economic value dropped because the harvest interval become too long. This enabled the fruit to become overripe and turn into waste.

By expanding the harvested maturity range to RS4–RS6, the effect of harvest interval was largely removed for the winter fruit. The reason for this being that winter fruit developed too slow to bridge the gap from RS3 to RS6, even during the 4 d intervals (Figure 7A).

Note that in both cases the economic value generated during the subsequent harvest days strongly depended on the harvest interval. Using 1 d intervals only small revenues were generated per harvest as only small amounts were harvested at once. By increasing the harvest interval, the early harvest blocks increased in size, but the latter ones were reduced. The reason for this being that during the early harvests the fruit was not yet developing at full speed (and therefore the first harvest could have been postponed) but during the latter harvests fruit is developing in average much faster and waste is being generated (shorter harvest intervals should have been applied to prevent waste). By adapting the timing of the harvest actions to the development of the crop overall revenue can be optimized. One example of such dynamic harvesting scenario is shown for winter fruit harvested at RS5 and RS6. It is clearly seen that the economic value generated during the dynamic harvest intervals was similar to that for 3 d harvest intervals while the labor cost was reduced due to less frequent harvests of the former than the latter.

The summer fruit was characterized by a faster fruit growth, affecting the outcome of the simulated harvesting strategies accordingly (Figure 7B). While the overall economic value remained lower as compared to the winter fruit, there was much more flexibility in improving the economic value by adapting the harvesting strategy. For the narrow maturity range (RS5 and RS6) the summer fruit showed an earlier decrease in economic value, starting from the 3 d harvest interval, as the fruit more rapidly turned into waste. For the wider maturity range (RS4, RS5, and RS6) the 1 d harvest interval resulted in a lower economic value as compared to the single harvest. This was due to the earlier start of the simulated harvesting season which, in combination with the frequent harvesting, resulted in an overrepresentation of relative small unripe fruit in all subsequent harvests as the fruit was not allowed to ripen properly. By increasing the harvest interval to 4 d revenues increased accordingly, in contrast to what was observed for the winter fruit. Similarly, a dynamic harvesting scenario was implemented for tomatoes harvested at RS5 and RS6. Even though the workload of the dynamic harvest was reduced by 30% compared to 2 d harvest interval (effectively 4 harvests under the dynamic scenario vs. 6 harvests under the 2 d harvest interval), it still generated the highest economic value compared to all fixed harvest intervals as it allowed the fruits fully develop and turn into good ripening stages with no mass going to waste (Figure 7B).

## Conclusions

The current study developed a population based approach to optimize the harvest strategy for “Savior” tomato grown in either winter or summer in Vietnam. Using the data on mass and color obtained during fruit development and ripening, a kinetic fruit growth model was successfully calibrated which then was used to quantify the population variation in terms of the physiological maturity of the tomatoes. While the applied model does not pretend to be a physiological model its level of detail seemed to be fit for the intended purpose of optimizing the postharvest economic value of the crop taking into account pre-harvest biological variation.

The calibrated growth model was successfully coupled to the wholesalers price model through a Monte Carlo approach to evaluate and optimize the harvest strategy with regard to economic value of the crop taking into account the omnipresent fruit-to-fruit variation. This study quantified an economic incentive for growers in developing countries to move away from their current single harvest strategy which will benefit the wider market by (*i*) spreading out fruit supply, (*ii*) increasing homogeneity of the fruit supplied to the market, and (*iii*) maximizing the profits for the growers and, above all, (iv) reducing post-harvest waste. It was shown that the potential sales value of a crop could be increased by undertaking multiple harvests assuming all other costs remain the same. The ideal situation was shown to depend on the rate of fruit development and ripening in relation to the choice of the targeted maturity range and the selected harvest interval. The total farm profit would still depend on other aspects such as different picking efficiencies at different crop densities, possible damage to the non-harvested crop or possible physiological effects on fruit development of the non-harvested crop by the reduced crop load, and the need for multiple transports. In a real application case the approach should be further detailed to align the timing of harvests with labor availability, market demands, available storage space, price uncertainty, etc. This work provides a first framework that allows the industry to design dynamic scenario's to start maximizing postharvest operations.

## Author contributions

DT, MH, and BN designed the experiments; DT, TT, NQ, CM, and BV acquired the experimental data; DT, MH, and CM analyzed the experimental data, all authors contributed to the interpretation of the data, DT and MH drafted the manuscript with all authors being involved in the revision of the manuscript.

### Conflict of interest statement

The authors declare that the research was conducted in the absence of any commercial or financial relationships that could be construed as a potential conflict of interest.
