# Supplementary material for: Population Modeling Approach to Optimize Crop Harvest Strategy. The Case of Field Tomato
Source: Front Plant Sci. 2017 Apr 20;8:608. doi: 10.3389/fpls.2017.00608 (PMC5397500; doi:10.3389/fpls.2017.00608)
Supplement: Supplementary file 1 [file Table1.PDF]

Supporting table

Table S1: Classification of the ripening stages for ‘Savior’ tomato based on Hue (º) limits

**Ripening stage class**	**Hue (º)**
RS1	> 106
RS2	98 - 106
RS3	83 - 98
RS4	69 - 83
RS5	60 - 69
RS6	54 - 60
Overripe	< 54
