# Supplementary material for: Population Modeling Approach to Optimize Crop Harvest Strategy. The Case of Field Tomato
Source: Front Plant Sci. 2017 Apr 20;8:608. doi: 10.3389/fpls.2017.00608 (PMC5397500; doi:10.3389/fpls.2017.00608)
Supplement: Supplementary file 2 [file Image1.PDF]

Supporting figure

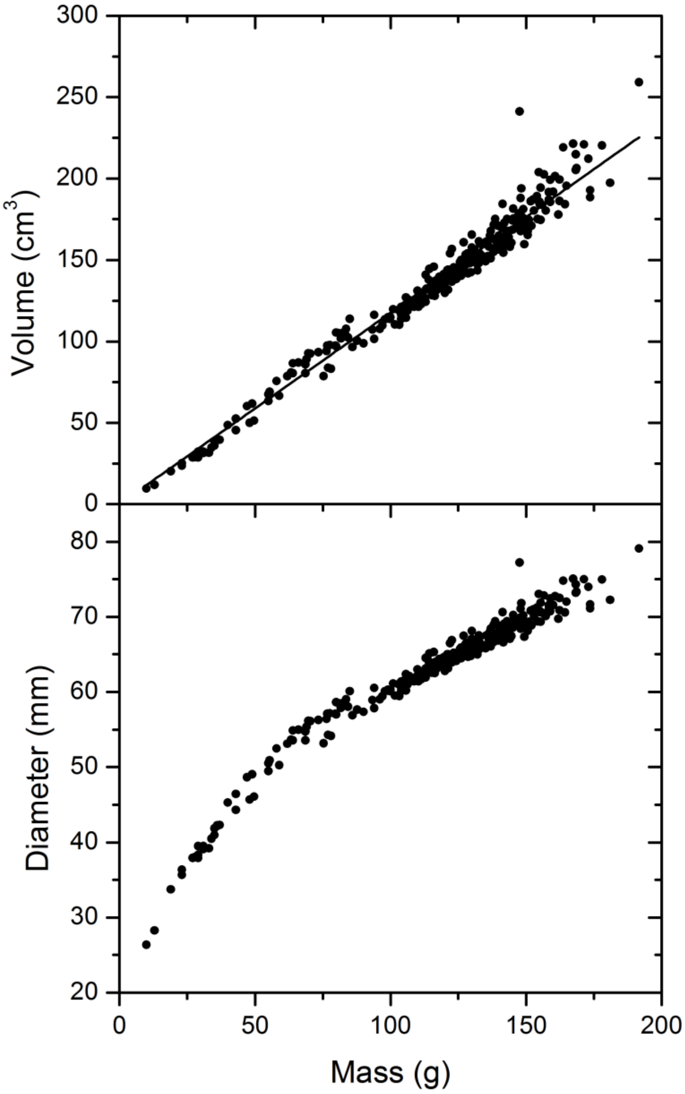

Fig. S1. Relationships between measured fruit mass and diameter and the calculated fruit volume as determined for 300 harvested tomato fruit. The straight relationship between fruit mass and calculated volume supports the assumption that fruit density can be considered constant for the developmental stages covering the range from 20-200 g.
