# Supplementary material for: Population Modeling Approach to Optimize Crop Harvest Strategy. The Case of Field Tomato
Source: Front Plant Sci. 2017 Apr 20;8:608. doi: 10.3389/fpls.2017.00608 (PMC5397500; doi:10.3389/fpls.2017.00608)
Supplement: Supplementary file 3 [file Image2.PDF]

Supporting figure

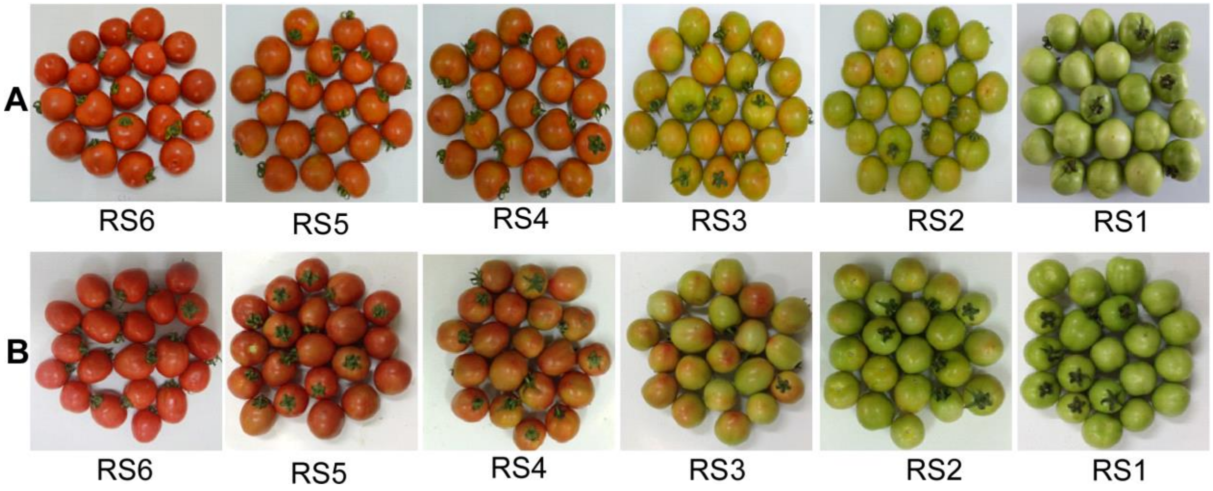

Fig. S2. Homogenous batches containing a single ripening stage (RS) of the tomato cv. ‘Savior’ grown A) in winter and B) grown in summer. RS1: mature green fruit, RS2: breaker fruit, RS3: light orange fruit, RS4: orange fruit, RS5: red fruit, RS6: red ripe fruit.
