# Supplementary material for: Population Modeling Approach to Optimize Crop Harvest Strategy. The Case of Field Tomato
Source: Front Plant Sci. 2017 Apr 20;8:608. doi: 10.3389/fpls.2017.00608 (PMC5397500; doi:10.3389/fpls.2017.00608)
Supplement: Supplementary file 4 [file Image3.PDF]

Supporting figure

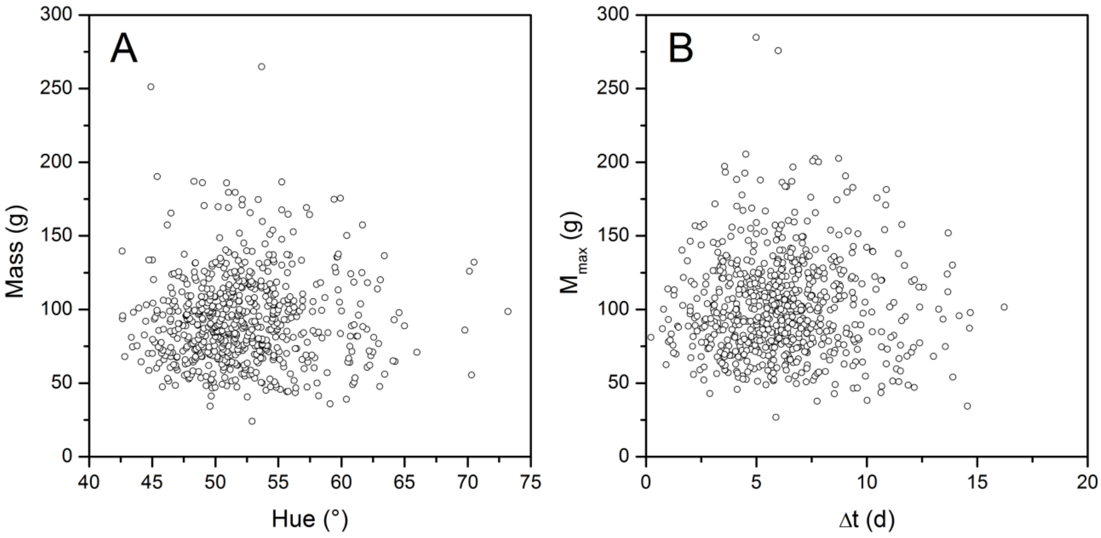

Fig. S3. Correlation plot for (A) the measured final hue colour and mass of the fruit and (B) the estimated fruit specific parameters M_{max} and the biological shift factor Δt.
